# The Elusive Origin of Atherosclerotic Plaque Calcification

**DOI:** 10.3389/fcell.2021.622736

**Published:** 2021-03-09

**Authors:** Emmanuelle Canet-Soulas, Laurence Bessueille, Laura Mechtouff, David Magne

**Affiliations:** ^1^CarMeN Laboratory, INSERM, INRA, INSA Lyon, University of Lyon, Université Claude Bernard Lyon 1, Lyon, France; ^2^ICBMS, CNRS, INSA Lyon, CPE, University of Lyon, Université Claude Bernard Lyon 1, Lyon, France; ^3^Stroke Department, Hospices Civils de Lyon, Lyon, France

**Keywords:** atherosclerosis, calcification, chondrocyte, inflammation, cardiovascular morbidity and mortality

## Abstract

It has been known for decades or even centuries that arteries calcify as they age. Vascular calcification probably affects all adults, since virtually all have atherosclerotic plaques: an accumulation of lipids, inflammatory cells, necrotic debris, and calcium phosphate crystals. A high vascular calcium score is associated with a high cardiovascular mortality risk, and relatively recent data suggest that even microcalcifications that form in early plaques may destabilize plaques and trigger a cardiovascular event. If the cellular and molecular mechanisms of plaque calcification have been relatively well characterized in mice, human plaques appear to calcify through different mechanisms that remain obscure. In this context, we will first review articles reporting the location and features of early calcifications in human plaques and then review the articles that explored the mechanisms though which human and mouse plaques calcify.

## Atherosclerotic Plaque Calcifications: The Smaller, the Scarier

### Different Types of Human Plaque Calcification

**FIGURE 1:**
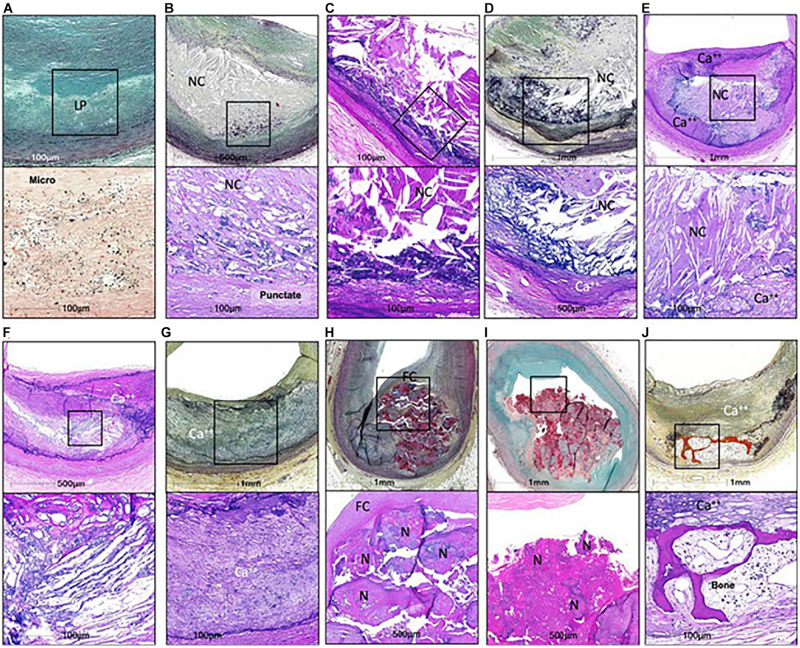
Calcifications in human coronaries. **(A,B,D,G,H,I,J)** Images in the top row are Movat pentachrome stained, and **(C,E,F)** Images are H&E stained; lower row shows high power image; corresponding to **(A)** Is Von Kossa staining and all others are H&E. Non-decalcified arterial segments **(A)** And all others are decalcified segments **(B–J)**. **(A)** Pathological intimal thickening characterized by a lipid pool (IP) that lacks VSMCs. Corresponding high power image (Von Kossa staining) of the boxed area shows microcalcification <15 μm in diameter within the LP. **(B)** Fibroatheroma showing an early necrotic core (NC) infiltrated by macrophages which are calcified, seen as punctate (≥15 μm) areas of calcification. **(C)** Fibroatheroma with a late NC and fragmented calcification seen toward the medial wall. **(D)** Late fibroatheroma with larger area of calcification occupying an area greater than 1 mm that shows calcification of the NC. **(E)** Fibrocalcific plaque with sheet calcification and calcifying NC, which is incompletely calcified. **(F)** Fibrocalcific plaque showing sheet calcium with both fibrous tissue and NC completely calcified. **(G)** Fibrocalcific plaque with sheet calcium without a NC. **(H)** Nodular calcification showing fragment of calcium separated by fibrin and lumina I coverage by fibrous cap. **(I)** Calcified nodule showing nodules of calcium within the lumen and an overlying thrombus. **(J)** Fibrocalcific plaque with an area of ossification at the edge of sheet calcification. Ca^++^, calcification; FC, fibrous cap; H&E, hematoxylin and eosin; N, nodule. From Jinnouchi et al. (2020), with permission from Elsevier.

Cardiovascular diseases are the leading cause of death worldwide (Roth et al., 2017). Atherosclerotic plaque rupture is the primary mechanism responsible for myocardial infarction and accounts for about 20% of cases of ischemic stroke (Ornello et al., 2018). Since coronary artery calcium scores correlate with cardiovascular mortality in asymptomatic individuals (Greenland et al., 2004), it was long believed that plaque calcification had a detrimental impact on plaque stability. In the last two decades, however, clinical and preclinical studies suggested that plaque calcification may have either beneficial or detrimental effects depending on the amount of calcium and type of calcification. Human plaques, in particular, can present very different types of calcification (Figure 1; Herisson et al., 2011; Jinnouchi et al., 2020). Microcalcifications, defined by size <15 or 50 μm depending on the author, can be observed in early type I lesions (Roijers et al., 2011; Chatrou et al., 2015). They sometimes coalesce to generate punctate calcifications with a size between 15 μm (or 50 μm) and 1 mm (Jinnouchi et al., 2020). Bigger calcifications comprise fragment calcifications, which measure more than 1 mm, and sheet calcifications, defined by size more than 3 mm (Jinnouchi et al., 2020). Nodular calcifications result from the fracture of calcified sheets under mechanical stress, such as that associated with coronary hinge motion (Lee et al., 2017). Finally, plaque ossification, with trabecular-like structures and bone marrow, is also sometimes observed although predominantly in peripheral arteries (Herisson et al., 2011). If macrocalcifications were historically considered to be harmful, a new paradigm has recently emerged, suggesting that heavily calcified plaques are in fact more stable. This paradigm relies in particular on the assumption that, with progressive calcification, plaque inflammation becomes pacified, and the necrotic core walled off from the blood (Dweck et al., 2016). On the other hand, biomechanical studies still indicate that macrocalcifications likely generate a significant mechanical stress that may negatively affect plaque stability [reviewed in Barrett et al. (2019)]. It is not our aim in this article to discuss the clinical impact of macrocalcifications but to describe the molecular mechanisms through which calcification is initiated in atherosclerotic plaques.

### Microcalcifications: New Suspects for Plaque Rupture

The discovery of microcalcifications, as opposed to large calcifications, in soft plaques undergoing pathology analysis (Vengrenyuk et al., 2006; Maldonado et al., 2012; Kelly-Arnold et al., 2013) suggested a role in acute events involving stress-induced plaque rupture, a mechanism long suspected in coronary arteries (Richardson et al., 1989). Microcalcifications are probably dangerous when they form in the fibrous cap (Vengrenyuk et al., 2006; Maldonado et al., 2012; Kelly-Arnold et al., 2013). Fibrous cap thickness is known to correlate with plaque vulnerability (Burke et al., 1997), and the presence of >5 μm microcalcifications might be particularly harmful, generating mechanical stress (Kelly-Arnold et al., 2013). In addition, microcalcifications exacerbate plaque inflammation, stimulating macrophages to release more tumor necrosis factor alpha (TNF-α) (Nadra et al., 2005, 2008), which also likely has detrimental effects on plaque progression.

While there is still no experimental proof that microcalcifications are indeed the most likely to trigger plaque rupture, an increasing number of studies using positron emission tomography (PET) with sodium fluoride 18 radiotracer (^18^F-NaF) have spotlighted the risk. Fluoride ions replace hydroxyl ions preferentially in newly formed, immature apatite crystals and are therefore a very interesting tool for detecting microcalcification (Hawkins et al., 1992). ^18^F-NaF PET has long been used to detect abnormal bone formation (Hawkins et al., 1992), but it was not until 2010 that a hybrid PET-CT camera enabled detection of ^18^F-NaF fixation in atherosclerosis plaque (Derlin et al., 2010, 2011). Seminal studies showed that ^18^F-NaF uptake was associated with features of instability and was more pronounced in clinically adjudicated culprit plaques in patients with myocardial infarction or ischemic stroke (Joshi et al., 2014; Vesey et al., 2017). However, it is now established that not only microcalcifications but also bigger ones that are actively growing are ^18^F-NaF positive, complicating the clinical interpretation of ^18^F-NaF PET-CT (Irkle et al., 2015; Høilund-Carlsen et al., 2020). Nevertheless, whereas the classical CT calcium score for macrocalcification is used to assess atherosclerotic load in coronary arteries, whole-body ^18^F-NaF PET-CT may be used as a marker of atherosclerotic plaque burden characterizing the patient’s global risk rather than focusing exclusively on the culprit plaque (Arbab-Zadeh and Fuster, 2015). In the clinical setting, PET-MRI will certainly help to better characterize the microcalcification process in plaque composition and will be more suitable for longitudinal studies. In a recent review on PET-MRI, Evans et al. (2020) pointed out the importance of combining ^18^F-NaF molecular calcification imaging and high-resolution MRI plaque characterization. An initial study by our group found that ^18^F-NaF-positive lesions were not associated with known MRI criteria of vulnerability (Mechtouff et al., 2020), but further longitudinal studies could helpfully use contrast-enhanced MRI to check whether ^18^F-NaF uptake precedes the vulnerable state. As longitudinal clinical studies are still lacking and are also rare in preclinical models of microcalcification (Hsu et al., 2020), the mechanisms underlying ^18^F-NaF uptake and the link with specific inflammatory processes are not fully elucidated. We are currently performing a preclinical study in mice with ^18^F-NaF PET to determine the impact of microcalcifications on plaque development.

If, in the near future, the harmful impact of microcalcifications is experimentally proven, it will be crucial to better understand how they form. Two models can be drawn from the literature. First, vascular smooth muscle cells (VSMCs) may undergo phenotypic changes to transform into osteochondrocyte-like cells. Many factors have indeed been shown to induce this phenotypic transformation. An alternative hypothesis is that calcification initiates independently of osteochondrocyte markers; this is supported by histopathological findings. In the next two chapters, we review the arguments in favor of each hypothesis.

## Arguments in Favor of the Phenotypic Change Hypothesis

### Plaques Are Calcified by Endochondral Ossification in Mice

Since it is obviously extremely difficult to analyze the longitudinal process of plaque calcification in humans, atherosclerotic mice have been widely used to investigate how plaques calcify. Two mouse models have been explored in depth: mice deficient in apolipoprotein E (ApoE) were used in most studies and mice deficient in low-density lipoprotein receptor (Ldlr) in some others. When *ApoE*-deficient mice are given a high-fat diet from 10 weeks of age, calcification can be detected in the aorta from the age of 20 weeks (Aikawa et al., 2007). Histological examination of animals aged between 45 and 75 weeks revealed the presence of chondrocyte-like cells, expressing type II collagen in calcified regions, suggesting that plaque calcification develops through a process mimicking endochondral ossification (Qiao et al., 1995; Rattazzi et al., 2005). Importantly, in these studies, the authors observed chondrocytes before calcifications and concluded that plaque calcification results from cartilage metaplasia (Rattazzi et al., 2005).

If plaque calcification is indeed an active process relying on chondrocyte differentiation, and if VSMCs are involved, then deletion of RUNX2 in VSMCs should prevent it. RUNX2 is the master transcription factor governing the differentiation and maturation of mineralizing cells, i.e., hypertrophic chondrocytes and osteoblasts (Komori et al., 1997). Among other transcriptional targets, RUNX2 stimulates the expression of tissue-non-specific alkaline phosphatase (TNAP), a promineralizing enzyme (Murshed et al., 2005). TNAP allows mineralization to occur by hydrolyzing inorganic pyrophosphate (PP_*i*_), a constitutive mineralization inhibitor (Hessle et al., 2002; Murshed et al., 2005). Mineralization is physiologically restricted to growth-plate cartilage and bone despite TNAP being relatively ubiquitous because mineralization requires a fibrillar collagen as a template for crystal deposition and because TNAP and fibrillar collagens are only coexpressed in growth-plate cartilage and bone (Murshed et al., 2005). Two independent groups produced and analyzed atherosclerotic mice deficient in *RUNX2* specifically in VSMCs. In *ApoE*^–/–^ mice, *RUNX2* deletion almost completely prevented TNAP expression and calcification (Sun et al., 2012). VSMC-specific deletion of *RUNX2* in *Ldlr*^–/–^ mice reduced plaque calcification, with a decrease in several RUNX2 transcriptional targets such as TNAP and type X collagen (Lin et al., 2016). These two articles clearly suggest that most calcium deposition in mouse plaque relies on VSMC phenotypic change into hypertrophic chondrocytes. Many factors have been shown to stimulate VSMCs to transdifferentiate into mineralizing cells, but the vast majority are associated with inflammation and oxidative stress, which often go hand in hand (Demer and Tintut, 2011).

### Plaque Ossification Appears to Be Stimulated by Inflammation in Mice

Interestingly, early calcification was associated with inflammation in the aorta of *ApoE*^–/–^ mice (Aikawa et al., 2007). A wide range of inflammatory molecules relevant to atherosclerosis have been shown *in vitro* to trigger the phenotypic change of VSMCs into RUNX2-expressing osteoblast-like cells and/or chondrocyte-like cells. Inflammatory phenotypic change cytokines such as TNF-α, interleukin (IL)-1β, and IL-6 are particularly potent calcifying factors in human and murine VSMCs [reviewed in Bessueille and Magne (2015)]. These promineralizing effects of inflammatory cytokines on VSMCs may not only be *in vitro* findings, since treatment of *Ldlr*^–/–^ mice with an anti-IL-1β (Awan et al., 2015) or anti-TNF-α (Al-Aly et al., 2007) antibody reduced plaque calcification. In addition, toll-like receptor (TLR)-2 and TLR-4 agonists such as lipopolysaccharide also stimulated VSMC change into chondrocytes *in vitro*, and *ApoE*^–/–^ mice also deficient in TLR-2 developed reduced plaque calcification with reduced cartilage metaplasia (Lee et al., 2019). This effect of LPS might be relevant to plaque calcification, since endotoxemia occurs after virtually all fatty meals (Herieka and Erridge, 2014), and is associated with atherosclerosis (Wiedermann et al., 1999).

This stimulatory effect of inflammation on VSMC phenotypic change into RUNX2-expressing chondrocytes is in contrast to the known inhibitory effect of inflammation on chondrocyte differentiation (Lencel et al., 2011). One possible explanation is that inflammation stimulates expression of bone morphogenetic protein 2 (BMP2), a strong bone anabolic factor, in VSMCs (Ikeda et al., 2012), which may induce VSMC differentiation into chondrocytes when inflammation begins to resolve (Li et al., 2008; Liberman et al., 2011). Interestingly, reduced plaque calcification in *Ldlr*^–/–^ mice with anti-TNF-α treatment was associated with reduced BMP2 levels (Al-Aly et al., 2007). In addition, VSMC-targeted overexpression of BMP2 in *ApoE*^–/–^ mice resulted in increased plaque calcification (Nakagawa et al., 2010). More importantly, inhibition of BMP2 with a small chemical inhibitor or with a recombinant BMP antagonist decreased plaque calcification, lipid deposition, and inflammation in *Ldlr*^–/–^ mice (Derwall et al., 2012). Furthermore, *ApoE*^–/–^ mice also deficient in BMP endothelial cell precursor-derived regulator (*Bmper*) exhibited increased BMP activity in endothelial cells and developed larger and more calcified atherosclerotic lesions (Pi et al., 2012). Finally, inhibition of plaque calcification by overexpression of matrix Gla protein (MGP), which is suspected to act as an inhibitor of BMP2 signaling (Zebboudj et al., 2002; Malhotra et al., 2015), decreased plaque calcification and lesion development in *ApoE*^–/–^ mice, in association with reduced BMP activity, strongly reduced macrophage infiltration and inflammation (Yao et al., 2010). Taken together, these results suggest that plaque calcification is dependent on BMP2 and therefore on chondrocyte differentiation. They also suggest that the development of calcified cartilage adversely impacts plaque development. This hypothesis, however, has to be considered with caution because chemical BMP inhibition reduced cholesterol biosynthesis and attenuated liver steatosis, suggesting that indirect effects might account for BMP2’s impact on plaques (Derwall et al., 2012). Moreover, MGP may inhibit vascular calcification independently of BMP2-stimulated VSMC change into chondrocytes (Leroux-Berger et al., 2011; Khavandgar et al., 2014), and MGP deficiency, which strongly increases vascular calcification, also protected against lesion development and inflammation in *ApoE*^–/–^ mice (Yao et al., 2010).

Similarly to pathogen-associated molecular patterns (PAMPs), such as LPS, and inflammatory cytokines, oxidized lipids may represent danger-associated molecular patterns (DAMPs), activating receptors in the toll-like receptor superfamily (Miller et al., 2011), and stimulate calcification in VSMCs culture (Demer and Tintut, 2011). Oxidative stress generated by H_2_O_2_ induced RUNX2 expression through AKT in mouse VSMCs (Mody et al., 2001; Byon et al., 2008). Acetylated low-density lipoproteins (LDLs) induced greater calcification than native LDL in human VSMCs (Proudfoot et al., 2002). In bovine VSMCs, oxidized LDL increased TNAP activity and calcification (Parhami et al., 2002). Oxidized LDL stimulated calcification in human VSMCs through TLR-4 and expression of osteochondrogenic factors (Song et al., 2017). Chondrogenic differentiation and calcification in response to oxidized LDL in human VSMCs involve activation of transforming growth factor (TGF)-β (Yan et al., 2011). These *in vitro* findings are, nevertheless, in contradiction with the recent report that VSMC-specific ablation of TGF-β signaling in *ApoE*^–/–^ mice leads to aortic aneurysms, with extensive lipid and calcium accumulation throughout the aorta (Chen et al., 2020); in this study, deletion of TGF-β signaling in VSMCs led to their dedifferentiation toward mesenchymal stem cells, enabling commitment toward chondrocytes and adipocytes (Chen et al., 2020). Finally, while inflammatory and oxidized molecules promote VSMC phenotypic change and calcification *in vitro*, how they act *in vivo* and whether they activate BMP and TGF-β growth factors still needs to be better understood. Moreover, there are solid arguments supporting the hypothesis that, *in vivo*, plaque calcification begins independently of VSMC phenotypic change.

## Arguments Against the Phenotypic Change Hypothesis

### Not So Many Human Plaques Calcify Through an Ossification-Like Process

While plaque calcification incontestably develops by a process similar to endochondral ossification in mice (Rosenfeld et al., 2000; Rattazzi et al., 2005; Lin et al., 2016), studies in humans showing the presence of chondrocytes or cartilage are rare to say the least and remain controversial (Tyson et al., 2003; Aigner et al., 2008; Kuzan et al., 2017). In addition, bone-like structures, regardless of whether they formed through endochondral or intramembranous ossification, are not the most common type of calcification in human plaques (Jinnouchi et al., 2020): they are frequently observed in femoral arteries but not, for instance, in carotids (Herisson et al., 2011). Furthermore, the increasing number of studies of microcalcifications in early plaques reported that they formed independently of chondrocyte- or osteoblast-like cells or markers. In coronary arteries, for instance, microcalcifications were observed in preatheroma type I lesions before BMP2, or the RUNX2 transcriptional target osteocalcin could be detected (Roijers et al., 2011; Chatrou et al., 2015). It seems unlikely that, in these studies, chondrocytes or osteoblasts were present and but not be detected because, in most cases, microcalcifications that had grown and coalesced generated macrocalcifications still devoid of osteoblast or chondrocyte activity (Herisson et al., 2011; Jinnouchi et al., 2020).

How can we explain that mouse plaques calcify through endochondral ossification, whereas human plaques more frequently develop independently of chondrocytes or osteoblasts? One explanation could be that, in mice as in humans, calcification initially occurs independently of chondrocyte differentiation, which is induced later on. It is noteworthy that, in mice, VSMC-specific deletion of RUNX2 reduces but does not fully prevent plaque calcification (Sun et al., 2012; Lin et al., 2016). It is, however, difficult to know whether calcifications that formed despite the absence of RUNX2 in VSMCs did so independently of RUNX2 or rather under the control of RUNX2 in cells other than VSMCs (Lin et al., 2016). In the next section, we will review the main mechanisms that may lead to calcification independently of RUNX2 and of chondrocytes or osteoblasts.

### Calcification May Begin on Cell Debris

One very plausible mechanism of early microcalcification formation involves cell debris (Kim, 1995). Whereas extracellular calcium phosphate precipitation is mainly prevented by the presence of mineralization inhibitors such as PP_*i*_, intracellular calcification is normally prevented by the physical separation of calcium ions and inorganic phosphate (P_*i*_). Calcium is mainly stored in the endoplasmic reticulum and is present at very low concentrations in the cytoplasm, where P_*i*_ levels are higher than in the extracellular fluids (Romero-Garcia and Prado-Garcia, 2019). Apoptosis normally does not compromise this separation, or at least does not allow intracellular calcification to propagate extracellularly, because integrity of the cell membrane and of the membrane of apoptotic bodies (ABs) is preserved. However, when ABs are not phagocytosed rapidly enough by macrophages, they undergo necrosis, characterized by membrane rupture, allowing calcium, and P_*i*_ to precipitate. It is well known that apoptotic debris clearance is impaired in atherosclerotic plaque (Schrijvers et al., 2005), and calcification on apoptotic debris is frequent in early plaques. Histological examination of thousands of coronary arteries revealed that microcalcifications often form in proximity to apoptotic VSMCs, and that calcifying apoptotic macrophages are often seen in association with punctate calcifications resulting from microcalcifications (Jinnouchi et al., 2020). The role of apoptosis in initiating calcification is strengthened by the report that inhibition of apoptosis by a broad caspase inhibitor reduced calcification in cultured human VSMCs (Proudfoot et al., 2000). Impaired AB clearance rather than ABs themselves is likely involved in calcification, since induction of apoptosis specifically in VSMCs *in vivo* does not lead to arterial inflammation or calcification in wild-type mice, whereas it leads to reduced fibrous cap thickness and collagen content, together with increased inflammation and calcification in *ApoE*^–/–^ mice (Clarke et al., 2006, 2008).

These data strongly suggest that necrosis secondary to VSMC apoptosis induces or increases plaque calcification. Alternatively, or in addition to secondary necrosis, programmed necrosis, also known as necroptosis, may participate in plaque calcification. Necroptosis was discovered relatively recently as a proinflammatory type of programmed cell death controlled by receptor-interacting serine/threonine-protein kinase 1 (RIPK1) and RIPK3 (Choi et al., 2019). Upon induction of necroptosis, RIPK3 phosphorylates the mixed-lineage kinase domain-like (MLKL) protein, which leads to MLKL oligomerization, membrane translocation, and formation of a pore allowing extracellular release of intracellular molecules (Kolbrink et al., 2020). Interestingly, *Ldlr*^–/–^;*Ripk3*^–/–^ mice develop smaller necrotic cores than *Ldlr*^–/–^ mice, indicating that necroptosis is a significant form of cell death in plaques, and exerts negative effects (Lin et al., 2013). This was confirmed in *ApoE*^–/–^;*Ripk3*^–/–^ mice, which showed less plaque inflammation and later mortality than *ApoE*^–/–^ mice (Meng et al., 2015). In addition, treatment of *ApoE*^–/–^ mice with necrostatin-1, an inhibitor of RIPK1–RIPK3 interaction and subsequent necroptosis, reduces lesion size and necrotic core formation (Karunakaran et al., 2016). Taken together, these studies suggest that necroptosis, like necrosis induced by impaired apoptotic cell clearance, has a detrimental effect in plaque development that might include induction of calcification. However, this interpretation, according to which necroptosis induces calcification and has harmful effects only in atherosclerosis, might be oversimplistic. Indeed, specific *Ripk3* deletion in macrophages or endothelial cells protects *ApoE*^–/–^ mice from lipid accumulation, suggesting that necroptosis impacts plaque development differently depending on the cell lineage in which it takes place (Colijn et al., 2020). Moreover, a recent study surprisingly showed that, while inhibition of MLKL expression in *ApoE*^–/–^ mice predictably impaired necroptosis, it increased lipid accumulation within the plaques (Rasheed et al., 2020). To our knowledge, whether plaque calcification can be prevented by inhibition of necroptosis has not yet been specifically investigated; since necrosis is known to induce calcification and since microcalcifications are often associated with dying macrophages and VSMCs (Jinnouchi et al., 2020), more studies are warranted.

In addition to necroptosis, another form of cell death, called pyroptosis, may participate in plaque calcification. Pyroptosis is the form of cell death associated with secretion of IL-1β relying on NOD-like receptor family pyrin-domain-containing 3 (NLRP3) activation (Kesavardhana et al., 2020). It has long been known that NLRP3 activation leads to caspase-1-mediated cleavage of pro-IL-1β into mature IL-1β; however, how this mature IL-1β is released extracellularly was discovered only recently. Activated caspase-1 not only cleaves the cytoplasmic protein gasdermin D, allowing the N-terminal fragment of gasdermin D to polymerize in the membrane and form pores through which IL-1β is released (Shi et al., 2015), but also leads to cell death (Kesavardhana et al., 2020). IL-1β is a very important cytokine in atherosclerosis, which modulates multiple aspects of plaque formation (Bhaskar et al., 2011) and development (Gomez et al., 2018) and has emerged as a promising target in patients with previous myocardial infarction (Ridker et al., 2017). However, probably because IL-1β has both beneficial and detrimental effects on atherosclerotic plaque development in *ApoE*^–/–^ mice (Bhaskar et al., 2011; Gomez et al., 2018), manipulation of caspase-1 and NLRP3 levels in *ApoE*^–/–^ mice provided contradictory results (Menu et al., 2011; Zheng et al., 2014; Yin et al., 2015; van der Heijden et al., 2017). To our knowledge, the possibility that pyroptosis participates in plaque calcification *in vivo* has not been specifically addressed, but *in vitro* inhibition of inflammasome activation reduced IL-1β secretion and inhibited VSMC calcification (Wen et al., 2013).

### Calcification May Result From the Release of Extracellular Vesicles and the Activation of TNAP

Finally, there are arguments suggesting that initiation of plaque calcification may be due to VSMCs that have acquired some functions of mineralizing cells, without truly differentiating into chondrocytes or osteoblasts. For instance, numerous *in vitro* studies and genetic models have shown that, often, the mere deficiency of a mineralization inhibitor or the mere upregulation of a promineralizing factor is sufficient to trigger calcification. In particular, several genetic diseases or mouse models indicate that a single enzyme, TNAP, is sufficient to induce arterial calcification. As described above, TNAP induces mineralization by hydrolyzing PP_*i*_ (Hessle et al., 2002; Murshed et al., 2005), and constant physiological production of PP_*i*_ is required to prevent vascular calcification. Deficient generation of PP_*i*_ from extracellular ATP, due to mutations in the gene encoding ectonucleotide pyrophosphatase/phosphodiesterase 1 (ENPP1), leads to a disease known as generalized arterial calcification of infancy (GACI) (Rutsch et al., 2003). Logically, VSMC-specific overexpression of TNAP is sufficient to induce medial calcification in mice (Sheen et al., 2015). Interestingly, calcification in this model is associated with increased transcript levels of the osteochondrocyte markers *Bmp2*, *Sox9*, *Acan*, and *Runx2* (Sheen et al., 2015), suggesting that TNAP not only stimulates calcification but also launches the whole phenotypic change process of VSMCs into osteochondrocyte-like cells. Molecular investigation of the mechanisms involved suggests that TNAP induces calcification in VSMCs, which in turn activates the bone anabolic factor BMP2 (Fakhry et al., 2017). Such a molecular sequence implies that TNAP is expressed before VSMC differentiation and independently of RUNX2. Another genetic disease, arterial calcification due to deficiency of CD73 (ACDC), offers a likely explanation (St Hilaire et al., 2011). CD73 is a relatively ubiquitous nucleotidase that dephosphorylates extracellular AMP into adenosine, to participate in the resolution of inflammation (Antonioli et al., 2013). Calcification in ACDC is due to upregulation of TNAP expression in the absence of adenosine, to compensate for decreased AMP dephosphorylation (Jin et al., 2016). TNAP has indeed recently been described as an anti-inflammatory nucleotidase, which explains its ubiquitous expression (Bessueille et al., 2020). Therefore, induction of TNAP expression in association with its inflammatory function may result in induction of plaque calcification, independently of RUNX2. The fact that TNF-α, IL-1β, or IL-6 stimulates TNAP expression in VSMCs supports this paradigm (Tintut et al., 2000; Shioi et al., 2002; Lee et al., 2010; Lencel et al., 2011; Zhao et al., 2012).

Finally, if TNAP emerges as a possible important contributor to microcalcification, it must be added that the role of TNAP in physiological mineralization is not to trigger crystal nucleation but to allow calcium phosphate crystals to grow (Fleisch et al., 1966; Millán and Whyte, 2016; Bottini et al., 2018). Crystal nucleation is thought to occur inside extracellular vesicles (EVs) released by hypertrophic chondrocytes and osteoblasts and generally named matrix vesicles (MVs) in the bone biology field (Bottini et al., 2018). Although still controversial, MVs are suspected to concentrate calcium and P_*i*_ through the channeling activity of annexins and Pit transporters, respectively (Yadav et al., 2016; Bottini et al., 2018). In addition, MVs may further concentrate P_*i*_ from phosphatidylcholine through the sequential activity of phospholipase A2, ectonucleotide pyrophosphatase/phosphodiesterase 6, and PHOSPHO1 (Roberts et al., 2007; Yadav et al., 2016; Stewart et al., 2018). Crystal formation inside MVs would then rely on phosphatidylserine-mediated nucleation (Wu et al., 1993; Cruz et al., 2020).

Increasing data suggest that VSMCs release EVs that may initiate vascular calcification similarly to the way MVs released by hypertrophic chondrocytes induce growth plate mineralization (Hutcheson et al., 2016). This suspected involvement of EVs in plaque calcification has been nicely reviewed recently (Kapustin and Shanahan, 2016; Bakhshian Nik et al., 2017; Blaser and Aikawa, 2018; Aikawa and Blaser, 2021). We will therefore briefly present what is known of their suspected contribution to plaque calcification. Electron microscopic exploration of human carotid plaques revealed that vulnerable plaques may contain more calcifying EVs than stable ones (Bobryshev et al., 2008). Interestingly, several distinct multilamellar vesicles were visible, suggesting that several types of vesicles may be involved in plaque calcification. In culture of VSMCs, calcification is reduced by inhibition of annexin A6 expression (Kapustin et al., 2011) or PHOSPHO1 activity (Kiffer-Moreira et al., 2013), two proteins thought to be important for MV-associated mineralization. However, there might be significant differences in the mechanisms governing EV and MV release and mineralization/calcification. It was particularly shown that, in VSMCs, sortilin regulates the load of TNAP into EVs and that sortilin deficiency reduces plaque calcification but not bone mineralization (Goettsch et al., 2016). Finally, not only MV-like EVs, which are membrane blebs but also exosomes, which have an intracellular origin, may be involved in VSMC-mediated calcification. Exosomes released from VSMCs were indeed shown to be enriched in factors involved in calcification, such as annexin A6 and phosphatidylserine (Kapustin et al., 2015; Kapustin and Shanahan, 2016). Finally and to add more complexity, macrophages have also been shown to release calcifying EVs (New et al., 2013; Aikawa and Blaser, 2021). Therefore, the origin and respective contribution EVs and exosomes to plaque calcification *in vivo* will be therefore a difficult task to assess, but which deserves intense efforts.

## Conclusion

Arguments in favor of the phenotypic change hypothesis mainly come from mouse models of atherosclerosis, whereas human studies rather suggest that calcification begins independently of osteoblast or chondrocyte differentiation (Table 1). If microcalcification in mice, like in humans, originates independently of chondrocyte differentiation, then it will be interesting to understand why microcalcifications always lead to ossification in mice, but so infrequently in humans. Apatite crystals stimulate mouse VMSCs *in vitro* to express BMP2 (Sage et al., 2011), which triggers their chondrocyte differentiation (Fakhry et al., 2017). Human coronary plaques express BMP2 in association with calcifications (Boström et al., 1993; Dhore et al., 2001; Chatrou et al., 2015). Whether and why BMP2 is less potent in humans deserves investigation. Finally, it cannot be excluded that several different mechanisms initiate plaque calcification, since microcalcifications can be seen in the necrotic core of human plaques as floating debris or in the fibrous cap (Jinnouchi et al., 2020).

**TABLE 1:** Arguments in favor of, and arguments against the hypothesis that calcification is initiated by osteochondrocyte-like cells.

**Hypothesis**	**Arguments supporting the hypothesis**	**References**
Plaque calcification is initiated by osteochondrocyte-like cells	Plaques calcify through endochondral ossification in *ApoE^–/–^mice*, with crystals observed by electron microscopy in proximity to chondrocyte-like cells	Qiao et al., 1995; Rattazzi et al., 2005
	Mouse and human VSMCs *trans-*differentiate in culture into osteochondrocyte-like cells in response to inflammatory and oxidative factors relevant to the context of atherosclerosis	Al-Aly et al., 2007; Awan et al., 2015; Bessueille and Magne, 2015; Lee et al., 2019
	VSMC-specific inactivation of RUNX2, the transcription factor governing hypertrophic chondrocyte and osteoblast differentiation strongly decreases arterial calcium content in *ApoE^–/–^* and *Ldlr^–/–^* mice	Sun et al., 2012; Lin et al., 2016
Plaque calcification is initiated independently from osteochondrocyte-like cells	VSMC-specific inactivation of RUNX2 strongly decreases, but does not totally prevent arterial calcium deposition in *ApoE^–/–^* and *Ldlr^–/–^* mice	Sun et al., 2012; Lin et al., 2016
	Human plaques only occasionally show bone-like structures at histology and evidence of endochondral ossification is lacking	Herisson et al., 2011; Jinnouchi et al., 2020
	Osteocalcin, a marker of hypertrophic chondrocytes and osteoblasts expressed under the control of RUNX2 is expressed after calcifications are formed in human plaques	Roijers et al., 2011; Chatrou et al., 2015
	Microcalcifications are often seen on VSMC and macrophage debris in human plaques	Herisson et al., 2011; Jinnouchi et al., 2020
	Inhibition of apoptosis decreases calcification in human VSMC cultures, and induction of apoptosis specifically in VSMC increases calcification *in ApoE^–/–^mice*	Proudfoot et al., 2000; Clarke et al., 2008; Clarke et al., 2006

## Author Contributions

All authors listed have made a substantial, direct and intellectual contribution to the work, and approved it for publication.

## Conflict of Interest

The authors declare that the research was conducted in the absence of any commercial or financial relationships that could be construed as a potential conflict of interest.
